# Medical Items

**Published:** 1896-04

**Authors:** 


### MEDICAL ITEMS.

THE heart bowed down by weight of woe,
The eyes that weep, the lips that quiver,
Are often largely due, we know,
To torpid liver. — *Washington Star.*

DR. J. H. GOSS, of Athens, will sail this month for Europe. We hope the doctor will send us some letters.

DR. C. H. TEBAULT, of New Orleans, succeeds the late Dr. Joseph Jones as Surgeon-General of the United Confederate Veterans.

DR. W. L. WRIGHT, formerly of Lost Mountain, Ga., has moved from Dundee to Brandon, Texas. He says, "Hurrah for the JOURNAL!"

DR. JAMES L. CAMPBELL has opened an office at No. 117 W. Mitchell St., where he will do a general practice. We wish him much success.

"MY books show that I have had a practice of $4,000 in the last year," said a physician. "Is your pocketbook included in the showing?" asked a bystander.

ACCORDING to the Indiana *Medical Journal*, W. R. Amick, of consumption-cure fame, is now a victim of consumption at Scipio, Ind. "He saved others; himself he cannot save."

THE American Medical Publishers' Association will hold its third annual meeting in Atlanta, Monday, May 4th. Important business will come before the association, and a large attendance is desired.

---

A TENNESSEE subscriber in renewing his subscription says: "The JOURNAL is the best monthly I have ever seen." Another stopped his subscription, and after three months, wrote to renew, asked for back numbers, and said he could not do without the JOURNAL.

---

NEVER use any ether for inhalation which has been left over. A fresh bottle should be opened for each operation. It is the deterioration of the ether by contact with air that is the principal cause of inflammation of the respiratory tract following its inhalation.—*Ex.*

---

THE "medical item" in our February issue, on top of page 770, should have been credited to the *Journal of the American Medical Association.* It is a felicitous thought well expressed, and all the honor rightly belongs to the bright editor of the above journal.

---

WITH the March issue the New Orleans *Medical and Surgical Journal* passes into the hands of Dr. Charles Chassaignac and Dr. Isadore Dyer. This is one of the oldest, most successful, and most deserving of Southern journals. We take pleasure in wishing it continued prosperity under the new management.

---

THE third International Congress of Dermatology will be held in London August 4th to 8th. The official program contains a number of important papers relating to skin diseases and syphilis. The American secretary of the congress is Dr. George T. Jackson, 14 East 31st St., New York, who invites correspondence from those wishing to attend.

THE directors of the Post-Graduate Medical School and Hospital have named one of their wards in memory of the late Dr. Charles Carroll Lee, who was for many years a professor in the institution. They have placed a tablet in the ward giving the names of those who combined to contribute the ten thousand dollars which was given for the purpose of the memorial. The faculty of the institution participated largely in the memorial gift.

---

WHAT shall the X-rays in photography be called? Already the suggestions are almost legion. Among these we note "Roentography," obviously objectionable; "electroskiography" and "electrography," which are unwieldy; "skiography," not so bad, but, according to the British *Medical Journal*, at least legitimate in structure and valid as to title; "radiography," "actinography," and "scotography" are inaccurate. Whoever suggested "shadowgraphy" should be cast into outer darkness.

---

THE American Medical Association meets in Atlanta, Ga., May 5th, and all delegates to this society should embrace the opportunity of visiting "Lookout Inn," a magnificent and historic spot on the crest of Lookout Mountain, overlooking Chattanooga, Tenn. A special invitation is extended to physicians to spend a week with us, *en route* to Atlanta, and a complimentary rate will be made for the occasion. Trains up the mountain make close connections in both the Central and Union depots, Chattanooga, running through to the Inn without change. For further information and handsome, illustrated booklet, address M. S. Gibson, Lookout Mountain, Tennessee.

---

THE much advertised "S. S. S." is said to be composed of fluid extract of smilax, sarsaparilla, fluid extract of stillingia sylvatica, fluid extract of lappa minor, fluid extract of phytolacca decandra

(each 16 parts), and tincture of xanthoxylum (8 parts); some nitrate of potash and a small quantity of iron are added, and enough alcohol to preserve it. "P. P. P." is made of fluid extracts of phytolacca, stillingia, sarsaparilla, xanthoxylum, with some compound tincture of gentian and iodide of potash. Radam's "Microbe Killer," is a mixture of impure sulphuric acid, four drams; impure muriatic acid, one dram; red wine, one ounce, with one gallon of water. Royal Germetuer is made by the addition of one pint of muriatic acid to a barrel of water. The Electropoise is composed of a nickel cylinder, to which is attached one end of a copper wire. The other end receives the confiding and deceived patient.

---

THE National Confederation of State Medical Examining and Licensing Boards will hold its fifth annual meeting in the Aragon Hotel, Atlanta, May 4th, at 10 o'clock. The objects of this conference are to discuss questions that pertain to State licensing with a view to a comparison and the improvement of methods, a collection and dissemination of information on the subject, and to consider any and all propositions that have for their object the advancement of the standard of medical education in the United States.

Dr. Chas. McIntire, of Pennsylvania, will read a paper on "Some Obstacles to an Interstate Recognition of a State License to Practice Medicine, with Suggestions for their Removal."

Dr. Jas. M. Mathews, of Louisville, Ky., and Dr. Wm. L. Foster, of Pittsburg, Pa., will also read papers.

The officers are: President, Dr. William Warren Potter, of New York; Vice-President, Dr. Jas. M. Hays, of North Carolina; Secretary and Treasurer, Benj. M. Griffith, of Illinois.

Members and ex-members of examining boards are specially invited to attend this meeting and take part in the discussions.

